# Personalizing HRI in Musical Instrument Practicing: The Influence of Robot Roles (Evaluative *Versus* Nonevaluative) on the Child’s Motivation for Children in Different Learning Stages

**DOI:** 10.3389/frobt.2021.699524

**Published:** 2021-10-01

**Authors:** Heqiu Song, Emilia I. Barakova, Panos Markopoulos, Jaap Ham

**Affiliations:** ^1^ Department of Industrial Design, Eindhoven University of Technology, Eindhoven, Netherlands; ^2^ Human-Technology Interaction, Department of Industrial Engineering and Innovation Sciences, Eindhoven University of Technology, Eindhoven, Netherlands

**Keywords:** child–robot interaction, robots in music education, motivation for musical instrument practicing, robots for personalized education, robot roles, learning stages

## Abstract

Learning to play a musical instrument involves skill learning and requires long-term practicing to reach expert levels. Research has already proven that the assistance of a robot can improve children’s motivation and performance during practice. In an earlier study, we showed that the specific role (evaluative role versus nonevaluative role) the robot plays can determine children’s motivation and performance. In the current study, we argue that the role of the robot has to be different for children in different learning stages (musical instrument expertise levels). Therefore, this study investigated whether children in different learning stages would have higher motivation when assisted by a robot in different supporting roles (i.e., evaluative role versus nonevaluative role). We conducted an empirical study in a real practice room of a music school with 31 children who were at different learning stages (i.e., beginners, developing players, and advanced players). In this study, every child practiced for three sessions: practicing alone, assisted by the evaluative robot, or assisted by the nonevaluative robot (in a random order). We measured motivation by using a questionnaire and analyzing video data. Results showed a significant interaction between condition (i.e., alone, evaluative robot, and nonevaluative robot) and learning stage groups indicating that children in different learning stage groups had different levels of motivation when practicing alone or with an evaluative or nonevaluative robot. More specifically, beginners had higher persistence when practicing with the nonevaluative robot, while advanced players expressed higher motivation after practicing with a robot than alone, but no difference was found between the two robot roles. Exploratory results also indicated that gender might have an interaction effect with the robot roles on child’s motivation in music practice with social robots. This study offers more insight into the child-robot interaction and robot role design in musical instrument learning. Specifically, our findings shed light on personalization in HRI, that is, from adapting the role of the robot to the characteristics and the development level of the user.

## 1 Introduction

Musical instrument learning appears to have collateral cognitive benefits (Hassler et al., 1985; Anvari et al., 2002; Hietolahti-Ansten and Kalliopuska, 1990). Compared with passively listening to music, the strongest benefits were reported to come from active music listening and music training (Rauscher and Hinton, 2006). For example, singing, learning to play a musical instrument, and recognizing and keeping pitches and beat could improve learner’s cognitive functions better than passive listening (Bernhard, 2002; Jausovec and Pahor, 2017). Due to the acquisition of a complex set of motor, sensory, and cognitive skills that learning a musical instrument requires (Lehmann et al., 2007) for beginners, it typically takes years to become skilled performers. Meanwhile, Ericsson et al. (1993) argue that the amount of deliberate practice is the major distinction between successful and unsuccessful learners through the long-term instrument learning process to reach a high-level achievement (Lehmann and Ericsson, 1997). Practicing at home is an important part of the instrument learning process for children (Hallam, 1998),but it is not an enjoyable activity for most children. It is crucial for teachers and parents to understand the significance of motivation in instrument learning, which is also a skill development process (Woody, 2004).

Besides practicing repeatedly, social factors also play an important role in the success of young children’s music lessons. Previous literature indicates that young children depend dominantly on extrinsic motivation (Zdzinski, 1996), and, for example, peer support and peer tutoring can motivate the learner to engage in practicing music vigorously (Burnard, 2002), parental involvement is also a key factor that influences young children’s persistence and achievement in instrument learning (Creech and Hallam, 2003; Moore et al., 2003). With the rapid development of social robots and the benefits of using robots in different educational contexts (Brown and Howard, 2014; Shiomi et al., 2015; Kennedy et al., 2016), social robots could also be used to provide social support to motivate children in instrument learning. And the main aim of using social robots in education is mainly to arouse children’s motivation and improve the outcome of learning.

Earlier research suggested that the presence of the robot influences children’s motivation and performance for learning activities (Riether et al., 2012). This can be explained by social facilitation theory (Triplett, 1898). More specifically, social facilitation theory (confirmed by earlier research, see, e.g., Zajonc (1965)) describes that people perform better at well-trained tasks when audience is present, while on new and complex tasks, people perform worse when they know they are being observed by other people (and robots) (Riether et al., 2012). In addition, several studies suggested that evaluation apprehension may be a necessary condition for producing social facilitation effects (e.g., Cottrell et al. (1968); Jones and Gerard (1967)), Sasfy also suggested that social facilitation only works under the condition of people trusting the audience to have the potential to evaluate (Sasfy and Okun, 1974). So it might not simply be the presence of a robot, but rather the role that it has in the social interaction that determines a user’s response to the robot. That is, when a robot is present in an interaction as an evaluator of the user’s behavior, the user’s performance might be influenced. In general, earlier research has already deployed the role of robots as a tutor (Kennedy et al., 2016), a peer (Balkibekov et al., 2016; Park et al., 2017), a learner (Sandygulova et al., 2020; Hood et al., 2015), or a mediator of the interaction (Barakova et al., 2015).

Confirming the importance of the robot’s role in stimulating music practice, in an earlier study (Song et al., 2020), we designed two robot roles (i.e., evaluative role and nonevaluative role) as companions for children’s music practice and tested the influence of practicing while being assisted by a robot with one of the two roles on children’s motivation and performance (Song et al., 2021). Results showed that children were more motivated and performed better with the nonevaluative robot.

However, as described above, social facilitation theory (Triplett, 1898) shows that for new and complex tasks, being observed by an evaluator might be detrimental for performance and motivation, but for well-trained tasks, being observed by an evaluator has positive effects on performance and motivation.

Especially in the domain of instrument learning, such evaluation seems important. That is, in the domain of instrument learning, a person’s self-concept (a person’s perception of themselves, see, Greenberg (1970)) may be more crucial than in other domains (Marsh et al., 2003). A person’s self-concept is generated from an early age and more personalized when the person grows older (Marsh et al., 1991), which indicates that a learner’s self-concept differs in different learning stages. And this perception can become more positive because of the evaluations from others or comparison with others (Greenberg, 1970; Bong and Clark, 1999; Lamont, 2011).

Still, in the domain of human-robot interaction, the effect of how evaluative a robot role is for children in different learning stages has not been established yet. Therefore, in the current study, we investigated whether children in different learning stages would have higher motivation when assisted by a robot in a different supporting role (i.e., evaluative role versus nonevaluative role). We conducted an empirical study in a real practice room of a music school with 31 children who were at different learning stages (i.e., beginners, developing players, and advanced players). In this study, every child practiced for three sessions, practicing alone, assisted by the evaluative robot, or assisted by the nonevaluative robot (in random order). We measured motivation by using a questionnaire and analyzing video data. We expected that children in different learning stages would have higher motivation with different robot roles (i.e., evaluative role versus nonevaluative role).

## 2 Related Work

### 2.1 Musical Instrument Learning and Self-Regulation

From 1920s, researchers already started investigating the nonmusical benefits of musical training (Earhart, 1920; Hassler et al., 1985; Hietolahti-Ansten and Kalliopuska, 1990; Anvari et al., 2002). Additionally, as a skill learning activity, instrument learning requires amounts of practice through a combination of sensory input and output. The quantity and quality of practice have been one of the most important focuses in instrument learning, as well as collaborative music performance; evidence showed that it takes over 10 years for experts to train their skills to the level of a master (Ericsson et al., 1993; Williamon and Valentine, 2000; Thresher, 1964). Lehmann and Ericsson (1997) also pointed out, compared to unsuccessful learners, successful musicians put more effort into deliberate practice, which is one of the main reasons they reached a higher level. Practice has always played an important role in children’s instrument learning; however, it is not enjoyable especially for young children (Hallam, 1998). In this case, parental involvement becomes quite important in children’s persistence and achievement in instrument learning (Creech and Hallam, 2003; Moore et al., 2003). Nevertheless, with the increase of practice time, involvement of parents could exert pressure on children. And with time, it could eventually bring tense to the relationship between parents and their children.

In the development of children’s instrument learning, their practice habits in different learning stages are varied; proper habits take years to develop. The result from a study by McPherson and Renwick (2001) showed that playing a music piece from the beginning to the end is one of the practicing strategies that most of the beginners applied. Parncutt and McPherson (2002) considered this as the lack of awareness of the mistakes they make, since beginners normally do not have the ability to identify and correct their own mistakes. After investigating the practice habits of musicians and learners, researchers have also paid attention to the myriad factors implicated in musical learning, especially in musical instrument practicing, which offered a crucial backdrop for self-regulation research in instrument learning context. Started with McPherson and his colleagues from the 1990s, researchers started to investigate the educational construct which was known as self-regulation. According to Zimmerman’s sociocognitive model of self-regulation, personal perceptions, efficacy, and environmental conditions are involved in self-regulated learning (SRL) (Zimmerman, 1989), which indicated that a social context or an environment is an important part of students’ SRL. However, as a self-regulated activity, few researchers have applied self-regulation for social robot design in musical instrument learning. And self-regulated learning is related to the expertise level (Varela et al., 2016); we chose to focus on children’s learning stages in this study.

### 2.2 Motivation in Instrument Learning

Since social support is a key component in self-regulated learning and practicing is an important but not enjoyable part of the instrument learning process for children (Hallam, 1998), keeping children motivated during instrument learning seems important for teachers and parents. Self-determination theory has mainly been used to explain motivation in instrument learning (Renwick and Reeve, 2012; MacIntyre et al., 2018). This theory proposes that people have three core psychological needs, which are autonomy, competence, and relatedness. These psychological needs will be satisfied to different levels (Deci and Ryan, 2000; Ryan and Deci, 2000). Furthermore, as a crucial factor that can influence motivation to participate in learning activities, a student’s self-concept has found to be stronger in music than in other domains (Marsh et al., 2003), which means in order to maintain the motivation to persist in instrument learning, a strong music self-concept is a crucial component for it. However, as children age, these beliefs tend to change. Younger students are more inclined to have positive achievement beliefs. As they grow older, they tend to become more realistic about how successful they will be (Wigfield et al., 1997). This could be explained by children’s development of self-concept and the evaluation or feedback from others (Ireson and Hallam, 2009). Hence, it is intriguing to study the influence of evaluation on learners in different learning stages with different levels of self-concept.

### 2.3 Social Robot for Educational Use

As promising tools in education, educational robots are becoming a popular research topic, since there are proof that showed using a robot in the educational context could improve teachers’ effectiveness and students’ learning motivation. (Johnson et al., 2003; Papert, 1993). With the rapid development of technology and social robotics research, a lot of challenges researchers faced had already been solved or can be taken care of soon. For instance, in instrument learning, with the combination of sensors and cameras in a robot or other devices, the robot should be able to detect and correct wrong postures. There is also a well-developed system that we can build in the robot to detect the mistakes and evaluate the pitch and tempo of the performance (Asahi et al., 2018). We believe that student’s musicality can be developed well with more accurate judgements and individual strategies from the robot. We also envisioned the role of robot in children’s instrumental learning mainly as a companion, which is able to provide professional help and social support in children’s daily practice with the capability of 24/7 availability, flawless memory, mistake detection, big data, professional music knowledge, different teaching strategies, and so on. In the future, the robot could also be used to provide music teachers help in the music lessons to offer each child suitable learning experience. Research has found that interacting with a tangible robot resulted in more engagement than interacting with a video (Xie et al., 2008). Various educational scenarios have been employed with a social robot, including knowledge learning [e.g., math (Brown and Howard, 2014), science (Shiomi et al., 2015)] and skills learning [e.g., music (Han et al., 2009) and language (Nielsen et al., 2008)].

**FIGURE 1:**
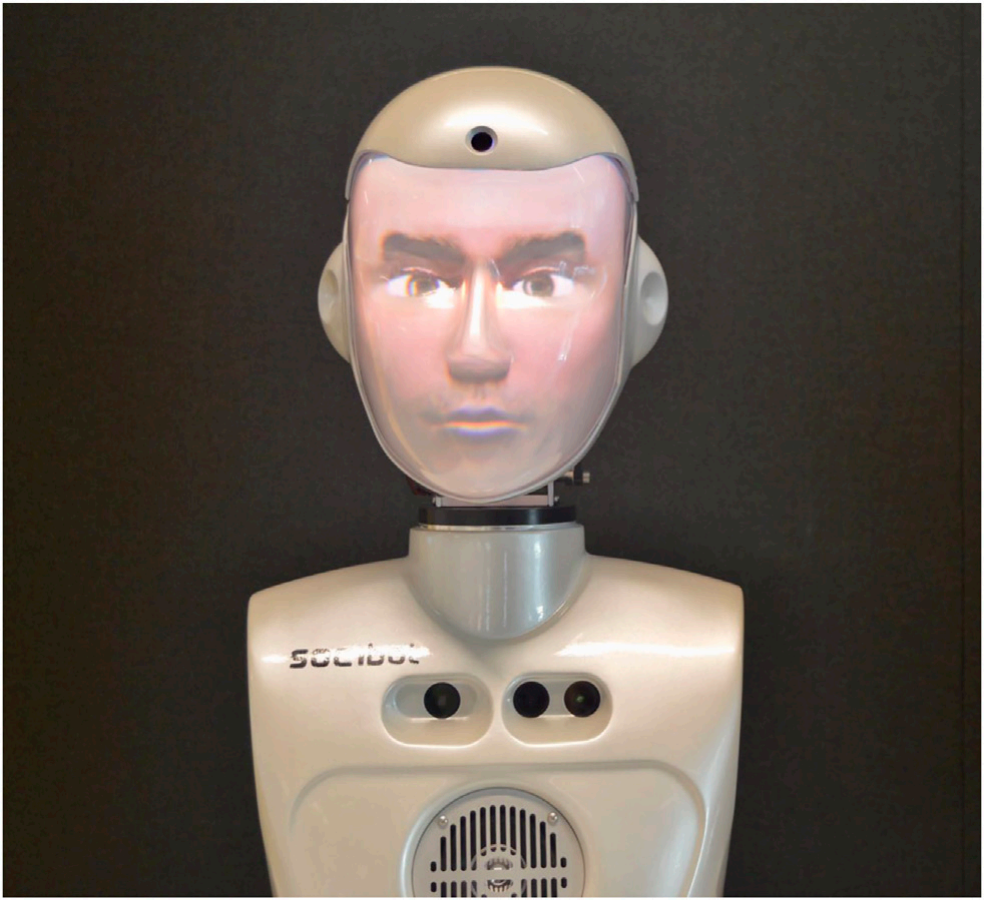
The SocibotMini robot used in the current study.

In educational scenarios, the roles of robots have also been explored. The robot can be deployed as a tutor (Kennedy et al., 2016), a peer (Balkibekov et al., 2016; Park et al., 2017), a learner (Sandygulova et al., 2020; Hood et al., 2015), or a mediator of the interaction (Barakova et al., 2015). By implementing the social facilitation theory and the evaluation apprehension theory, in the previous study (Song et al., 2020), we developed two roles (i.e., evaluative role and nonevaluative role) in the context of musical instrument practice by using SocibotMini (see Figure 1), which is also the robot we used in the current study, a robot with a projected face that provides rich human-like expressions. In the previous study, we performed the evaluation of those two roles in a real practice room at the music school. The study concluded that the designed evaluative and nonevaluative role of the social robot was convincing and matched children’s cognitive expectations in the music practicing context (Song et al., 2020).

### 2.4 Research Questions and Hypotheses

To figure out the impact of robot roles (i.e., evaluative role and nonevaluative role) on the motivation of children in different learning stages, we proposed the following:

• Research Question: Can different robot roles (i.e., evaluative role and nonevaluative role) affect the motivation of children in different learning stages differently in instrument practice?
• Hypothesis: We expect to find an interaction between the learning stage and robot condition (i.e., alone vs evaluative role vs nonevaluative role) on children’s motivation in instrument practice.

Furthermore, we will explore whether other factors influence the impact of different robot roles on children’s motivation in instrument practice.

## 3 Method

### 3.1 Research Context and Participants

This study was conducted at a common piano practicing room of a music school (Centrum voor de Kunsten Eindhoven, CKE) in the Netherlands. We chose the piano because the piano is one of the most popular musical instruments with children. In addition, it is quite easily possible to match the difficulty level of a new melody with the music learning level of a child, and also because of practical reasons (e.g., number of available potential participants at the local music school).

**FIGURE 2:**
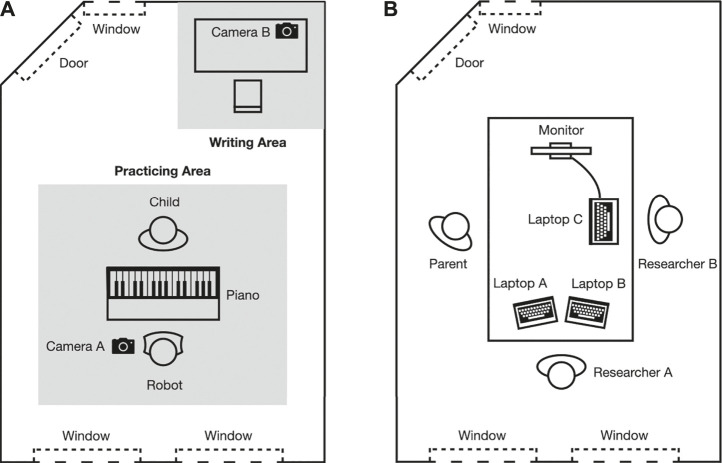
Top view illustration of the experiment room **(A)** and the control room **(B)**.

Two rooms were used for the experiment: an experiment room (see Figure 2A) for the participants to practice and interact with the robot and a control room (see Figure 2B) for the researcher to observe the process and control the robot. Two cameras (camera A and camera B) were set up in the experiment room before the experiment started. In the control room (see Figure 2B), researchers and parents could watch the child and the robot in the experiment room via the monitor which showed the view of camera B. We used the Wizard of Oz method to ensure situation-specific utterances of the robot. Researcher A in the control room monitored the experiment on laptop A (with the view of camera A in the practicing room) and the monitor (showing the view of camera B in the practicing room) to control the robot through laptop B. Camera A recorded the whole practicing session of each child.

The participants were 31 children (*N* = 31) aged from nine to 12 years old who were taking piano lessons in the music school. The learning stage of children, which indicates their level of expertise, ranged from 2 months to 5.5 years. Children were divided into three different learning stage groups based on the suggestion from piano teachers, which are beginners (had learned piano for less than 2 years, *n* = 11), developing players (had learned piano for two to 4 years, *n* = 10), and advanced players (had learned piano for more than 4 years, *n* = 10). The number of girls (*n* = 15) was approximately equal to that of boys (*n* = 16).

### 3.2 Robot Roles

We employed the two robot roles that we designed and evaluated in the previous study (Song et al., 2020). The evaluative role of the current study used “forceful and concrete language, providing praise on effort, with a slow and steady pitch, and a calm facial expression, focusing on the practice, move little, and dress formally (i.e., shirt)” (Song et al., 2020). In contrast, the robot in the nonevaluative role used “indirect and abstract language, provided praise on talent, with a quick and active pitch, and a funny facial expression, moved a lot, and dressed informally (i.e., striped sweater)” (Song et al., 2020). In the previous study, the interaction scripts for each role of the robot included 36 behaviors (Song et al., 2020). In the current study, children needed to practice two melodies in each session. To measure the performance data correctly, it is better if children can keep practicing one melody first and then change to the other one. In this case, we designed six new behaviors for the robot to indicate the melody the children should practice during practicing sessions. We also deleted some behaviors that were proved not suitable in the practicing context to simplify the interaction, but still kept the robot roles clear enough. In total, there were 30 behaviors of the robot adapted and designed (see Table 1).

**TABLE 1:** Numbers of robot behaviors for evaluative role and nonevaluative role in different tasks.

Context	Tasks	Evaluative robot	Nonevaluative robot
Robot introduction	Robot self-introduction	1	1
	Greeting	2	2
	General task introduction	3	3
Practice Session	General task introduction	1	1
	Melody order guide	2	2
	Verbal feedback for praise	3	3
	Verbal feedback for stop	1	1
	Verbal feedback for playing wrong melody	1	1
	Verbal feedback for questions	3	3
	Conclusion of the practice	1	1
Filling in questionnaire	Ask to fill in	2	2
	Ask to take a break	1	1

In addition, for the purpose of eliminating the ‘novel effect’ of practicing with a social robot, each child went through an introduction session with a robot, which has a neutral role. Twelve behaviors of the robot (i.e., a robot introduces itself and introduces the practicing task generally) were employed here in the introduction session to generate a fluent conversation (see Table 1).

### 3.3 Measures

We adapted and combined three subscales (i.e., autonomy, delight, and stress) from the FunQ questionnaire (Tisza and Markopoulos, 2021) and three questions from The Situational Motivation Scale (SIMS; Guay et al. (2000)) to measure the children’s motivation (see Table 2). The combined questionnaire consisted of four dimensions of motivation: autonomy, delight, stress, and interest. The FunQ questionnaire was developed to measure the fun value of a learning activity with adolescents around 12 year old, which is positively correlated with engagement (Iten and Petko, 2016; Rambli et al., 2013). We took the autonomy, delight, and stress dimensions from it as indicators of motivation. As for the interest dimension of the questionnaire, the questions in this dimension were adapted from the SIMS, in which questions were not included in the three dimensions mentioned before. Since the SIMS was not designed for children, based on the findings of Mellor and Moore (Mellor and Moore, 2014), the questions were changed into 5-point Likert questions and made easier by simplifying the language to the level of the children. Each dimension had three questions. Some questions had to be reverse coded: one question for the autonomy dimension and all three questions in the stress dimension were reversed questions that needed to be analyzed oppositely (Table 2). The motivation was also measured with observation data from the videos from camera A in Figure 2A.

**TABLE 2:** Motivation questionnaire questions.

Source	Dimension	Question
FunQ	Autonomy	I knew what to do
	—	I did this activity because I had to. (r)
	—	I did this activity because I wanted to
FunQ	Delight	I was happy
	—	I had fun
	—	I want to do something like this again
FunQ	Stress	I felt angry. (r)
	—	I felt sad. (r)
	—	I felt bad. (r)
SIMS	Interest	I could focus easily
	—	I think this practice is important
	—	I did this activity because I wanted to

r: reversed items.

### 3.4 Procedure

The participants were invited to the study through emails, and teachers from CKE helped us by sending the emails to the parents. After the participants and their parents arrived at the assigned room in the music school, they were asked to sign the informed consent form of the current study and fill in the questionnaire to collect basic information (e.g., gender, age, and learning duration). Then, researcher B explained the procedure in detail to them and escorted participants and their parents into the experiment room (see Figure 2A). In the meantime, the robot started to greet the children and encouraged them to talk to them. Next, the parents were invited to the control room while every child was practicing piano in three conditions (i.e., alone, with the evaluative robot, and with the nonevaluative robot) in random order, in a within-subject experiment design. In each condition, children needed to practice for 10 minutes (5 minutes for each music piece). At the beginning of the robot conditions, the robot gave a self-introduction first, including the name (i.e., “Jimmy” is the evaluative robot and “Peter” is the nonevaluative robot). After each session, they were asked to fill in the questionnaire to measure their motivation. And between each session, children were allowed to take a 5-minute break.

The melodies we chose to use in the experiment are parts of Chinese children’s songs because we want to make sure these are totally new to the (Dutch) participants. Eventually, we had in total nine pieces for each of the learning stage groups (i.e., beginners, developing players, and advanced players) and conditions (i.e., alone, evaluative robot, and nonevaluative robot). For instance, piece one is for beginners to play in the alone condition, piece two is for beginners to play in the evaluative robot condition, and piece four is for developing players to play in the alone condition. All the pieces were selected by a music teacher who has more than 25 years of experience of teaching the piano. The difficulty levels of the selected melodies were super easy for beginners (can be played by a single hand), easy for developing players (starting level of add another hand, for instance, at the start of each bar), and medium for advanced players (played by two hands, multiple keys at the same time). The duration of all the pieces was around 15 s since we only offered 5 min for them to practice each piece.

Regarding to the complexity of children’s motivation in instrument learning, our main purpose of this study is to test the different effects of different robot roles on children’s motivation. During the whole experiment, we kept all the possible confounding variables the same in the three conditions. The only difference between conditions is the type of accompanying (i.e., none (alone), evaluative robot, and nonevaluative robot) we provided. And thereby, we control for the influence of any other variable.

### 3.5 Data Analysis

The questionnaire data and video data were used to address whether children in different learning stages would have higher motivation with robots in different supporting roles (i.e., evaluative role versus nonevaluative role) in musical instrument practicing. First of all, by averaging the answers from the twelve questions, we were able to construct reliable measures for each dimension of the motivation scale, that is, autonomy (*Cronbach’s alpha* = 0.65), delight (*Cronbach’s alpha* = 0.80), stress (*Cronbach’s alpha* = 0.73), interest (*Cronbach’s alpha* = 0.77), and the questionnaire in total (*Cronbach’s alpha* = 0.89). To ascertain the impact of different roles of the robot on children’s motivation in practice, we divided the children into three different learning stage groups based on the suggestion from piano teachers. Additionally, as a nonempirical method, length of experience has been used as one of the factors to identify expertise in the clinic context (Rassafiani, 2009). The learning stage groups are beginners (had learned piano for less than 2 years), developing players (had learned piano for two to 4 years), and advanced players (had learned piano for more than 4 years). After that, we performed a repeated measure ANOVA (see Results section). Additionally, to increase the reliability and interpretability of the measurement for children’s motivation, despite the questionnaire, the video of children we took during the practicing was also analyzed. Because our video recordings (taken by camera A in Figure 2A) of the task were primarily focused on children’s faces and upper bodies, we coded the video data by using a validated coding scheme for assessing motivation in young children developed by Berhenke et al. (2011). They explored the importance of emotions in young children’s motivation, which they coded through facial movements (Izard, 1971). Meanwhile, they also took mastery motivation and strategy use as observed indicators of motivation. Eventually, they developed a coding system including four categories of codes, which were emotion states (neutral, positive, interest/arousal, sadness, confusion, anxiety, and anger), emotion events (pride, frustration, hostility, and shame), task behavior states (persistence, socializing and off-task), and task behavior events (help-seeking and competence). We adapted their coding system to fit the context of children’s piano practicing with the social robot by deleting codes that are not important in this context, adding behaviors that especially exist in musical instrument practicing, and adding codes to observe children’s interaction with the robot (Perugia et al., 2018). Finally, we analyzed the video data with the codes listed in Table 3.

**TABLE 3:** Descriptions for Emotion and Task Behavior Codes.

Category	Positive codes and indicators	Negative codes and indicators
**Emotional expressions**	**Happiness**	**Sadness**
	∗Laughs/giggles	∗Sad Expression
	∗Grins/smiles	∗Self-frustration
	∗Pride	**Anxiety**
	—	∗Anxious expression
	—	∗Shame/gaze avoidance/face hidden
	—	∗Confusion/frozen expression
**Task-related behaviors**	**Persistence**	**Hostility/Reluctance**
	-Visual focus point on task	∗Stop playing
	∗Showing initiative	∗Lean away from piano/leave
	∗Application of personal strategies	∗Shortcuts
	—	∗Frustrated verbal remarks
	**Help-seeking**	**Off-task**
	∗Verbally, directly, and explicitly ask for help	∗Signs of boredom
	—	-Visual focus off task
**Robot-related behaviors**	**Interest**	**Hostility/Reluctance**
	-Visual focus on robot	∗Refuses/hesitates/ignore to follow robot’s directions
	∗Engaged with a robot	∗Verbal remarks “must I really”
	∗Curiosity toward robot	—
	∗Reply to the robot positively	—
	∗Follows instructions	—
	**Help-seeking**	**Socializing**
	∗Chats with the robot about the task	∗Chats about other topics but the task

*Dichotomous nominal code.

*Continuous code.

Emotional expressions, task-related behaviors, and robot-related behaviors were coded independently by two independent researchers using the Observer XT 15.0 software system (Zimmerman et al., 2009) (*Cohen’s k* = 0.673, *p* < 0.01). Except for the persistence and off-task codes in the task-related behaviors category, which was coded continuously and analyzed by the percentage of focusing time in each mutually task-related behavior, the rest codes in the coding system were dichotomous nominal codes, which were counted and those counts were used in the analysis. Facial, vocal, and behavioral cues were used to indicate emotional expressions (e.g., triangular brows for sadness and smile for happiness) and task-related behaviors (e.g., intently keep practicing). Afterward, we performed independent sample *t* test to examine the impact of robots on children’s motivation and offer an additional explanation to the results of questionnaire data.

## 4 Results

In order to investigate the impact of robots in different supporting roles (i.e., evaluative role versus nonevaluative role) on the motivation of children in different learning stages in musical instrument practicing, the following analyses were performed. For the questionnaire data, firstly, we tested the normality of all questions in the questionnaire by using the Kolmogorov–Smirnov test and Shapiro–Wilk test. Results indicated that the answers to each dimension and question follow a normal distribution (*p* < 0.05). Then, we performed repeated-measures ANOVA to examine the impact of robot roles on children’s motivation (RQ). Results showed a main effect of the practicing condition (i.e., alone, with the evaluative robot and with the nonevaluative robot) on the children’s motivation measured using the questionnaire (*F* (2, 29) = 4.14, *p* = 0.02). Pairwise comparisons between the conditions showed that children had higher motivation after interacting with the evaluative robot (*M* = 4.53, *SD* = 0.77) and with the nonevaluative robot (*M* = 4.55, *SD* = 0.37) than after practicing alone (*M* = 4.34, *SD* = 0.56), with both *p*’s < 0.05.

Furthermore, confirming our first hypothesis, on *motivation as measured using the questionnaire*, results showed an interaction effect between robot role conditions (i.e., alone, with the evaluative robot, and with the nonevaluative robot) and learning stage groups [*F* (2, 29) = 2.88 and *p* = 0.03]. This indicated that children in different learning stage groups had different levels of motivation in the three conditions. More specifically, as shown in Table 4, compared to beginners (*M* = 4.40 and *SD* = 0.40) and developing players (*M* = 4.56 and *SD* = 0.39), advanced players showed the tendency to have the lowest motivation in the alone condition (*M* = 4.07 and *SD* = 0.97), as indicated by *t* = 1.69 and *p* < 0.10. For the two robot conditions (i.e., the evaluative role and nonevaluative role), such differences in motivation as measured by the questionnaire were not found, with both *t*’s < 1.00.

**TABLE 4:** Mean and standard deviation of motivation collected by the questionnaire in the alone, nonevaluative robot, and evaluative robot conditions.

	Alone			Nonevaluative robot			Evaluative robot		
	*n*	*M*	*SD*	*n*	*M*	*SD*	*n*	*M*	*SD*
Beginners	11	4.40	0.40	11	4.50	0.42	11	4.43	0.29
Developing players	10	4.56	0.39	10	4.63	0.33	10	4.53	0.43
Advanced players	10	4.07	0.97	10	4.53	0.39	10	4.65	0.56
Total	31	4.34	0.65	31	4.55	0.37	31	4.53	0.77

Additionally, we explored the *behavioral measures of motivation* and found comparable results on one of the indicators of motivation: persistence, which is very clearly an indicator on the participant’s motivation in instrument learning; see Table 5. For this, just as for the questionnaire data, we tested the normality of all the codes and the results showed that except for the interest in the ‘robot-related behaviors’ category, which is not calculated here in the comparison of motivation on task, all observational data follow a normal distribution (*p* < 0.05). On these data, we performed independent sample t tests. Confirming our finding on *motivation as measured using the questionnaire*, these analyses showed that advanced players (*M* = 2.80 and *SD* = 2.39) had lower persistence than beginners (*M* = 4.71 and *SD* = 2.43) and developing players (*M* = 3.67 and *SD* = 3.57) in the alone condition [*t* (2, 29) = 2.12 and *p* = 0.04]. It also showed that beginners’ persistence (*M* = 3.00 and *SD* = 2.94) was significantly higher than the advanced players (*M* = 1.90, *SD* = 1.60) in the nonevaluative condition [*t* (2, 29) = 4.03 and *p* = 0.00]. Furthermore, beginners also had higher persistence than developing players and advanced players, as indicated by a significant trend [*t* (2, 29) = 1.90 and *p* = 0.07]. For the evaluative robot conditions, such differences in motivation as measured by behavioral measures of motivation were not found, *t* < 1.00.

**TABLE 5:** Mean and standard deviation of motivation measured as persistence collected by the behavior data in the alone, nonevaluative robot, and evaluative robot conditions. Videos with a bad quality were not analyzed (incomplete, too much noise, etc.).

	Alone			Nonevaluative robot			Evaluative robot		
	*n*	*M*	*SD*	*n*	*M*	*SD*	*n*	*M*	*SD*
Beginners	7	4.71	2.43	7	3.00	2.94	7	3.57	1.51
Developing players	9	3.67	3.57	9	2.11	2.57	9	2.33	3.24
Advanced players	10	2.80	2.39	10	1.90	1.60	10	0.90	1.10
Total	26	3.62	2.86	26	2.27	2.31	26	2.12	2.36

After we compared the difference between learning stage groups in each of the conditions, we performed a comparison within each of the learning stage groups as well. We conducted paired sample *t* test on *motivation as measured by the questionnaire* within learning stage groups, and the results showed that advanced players had higher motivation in both robot conditions (i.e., evaluative role (*M* = 4.65 and *SD* = 0.56) and nonevaluative role (*M* = 4.53 and *SD* = 0.39)) than the alone condition [*M* = 4.07, *SD* = 0.97, *t* (2, 29) = 3.39, and *p* = 0.01; *t* (2, 29) = 2.02 and *p* = 0.07]. For the other two learning stage groups (i.e., beginners and developing players), such differences in motivation as measured by the questionnaire were not found, with both *t*’s < 1.00.

**FIGURE 3:**
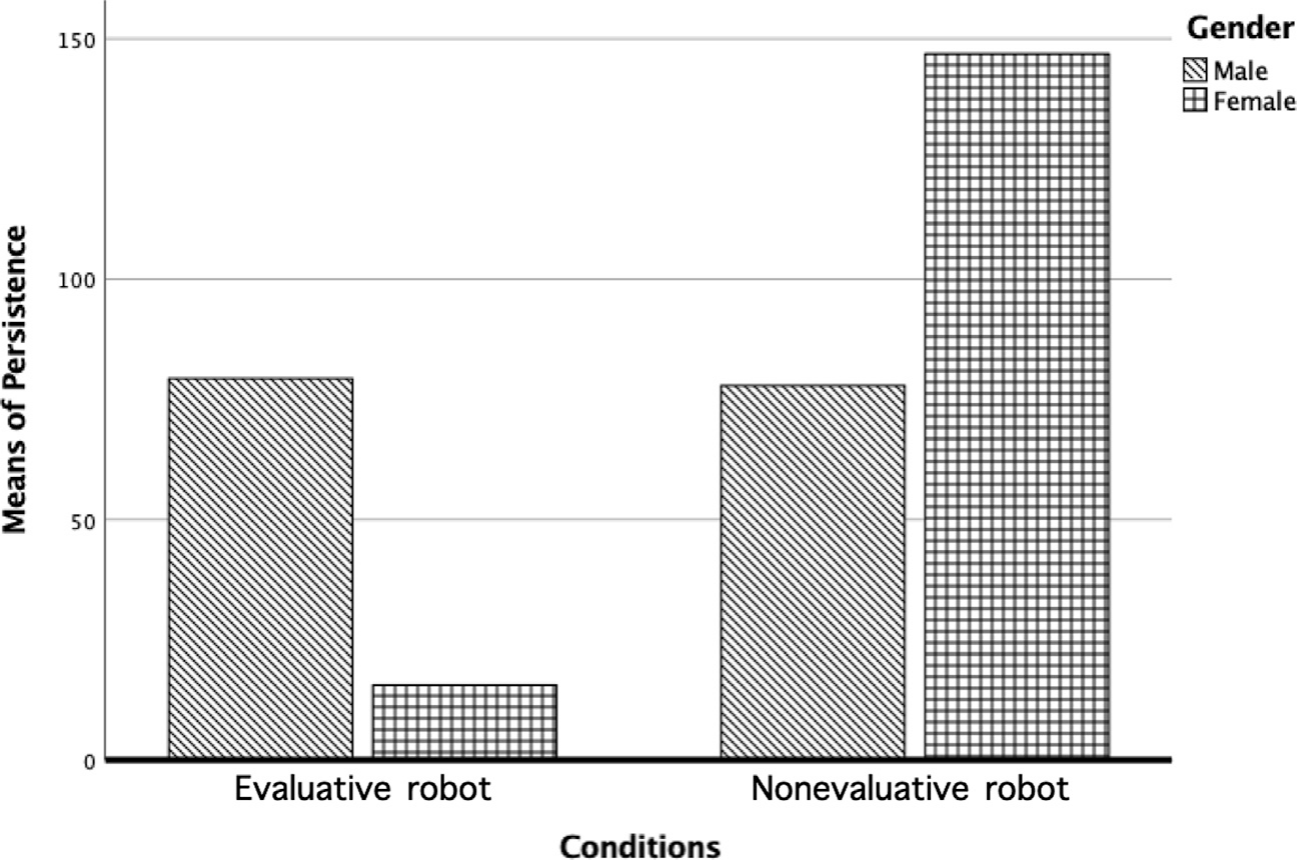
Means of persistence of children in different gender groups in the evaluative robot condition and the nonevaluative robot condition.

Furthermore, to explore other factors that may affect children’s motivation in music practicing with different roles of the robot, we also analyzed the difference between genders. According to the result we got from MANOVA, gender might also be a factor that influences children’s motivation through persistence in music practice [*F* (1, 30) = 5.02, *p* = 0.03]. As shown in Figure 3, under the nonevaluative robot condition, girls tended to have better persistence than boys, which means they focus on practice longer and follow more instructions. On the opposite, girls tended to persist less than boys in the evaluative robot condition while boys’ persistence level remained similar in both of the conditions. Except gender, we also checked whether age is the factor that influences children’s preference of the robot roles. Therefore, we conducted a correlation between the learning duration and age. Finally, the result showed no significant correlation between the learning stage and age (*r* = -0.01, *p* = 0.96), which suggested that the age of children did not influence the results we found on children’s learning stages. Also, this indicates that although younger people might have liked more the nonevaluative robot, younger people were not necessarily beginners.

## 5 Discussion and Conclusion

The aim of the current research was to find out the impact of robots in different supporting roles (i.e., evaluative role versus nonevaluative role) on children’s motivation in different learning stages in musical instrument practice. First of all, we performed a repeated-measure ANOVA to answer the first research question (RQ): Can robot roles affect the motivation of children in different learning stages differently? The main effect firstly indicated that regardless of the learning stages children in, children tended to have higher motivation with the nonevaluative role. Then, the result of the interaction effect combined with the result from behavioral data showed differences between different learning groups, which could answer the research question. With the result we got from the questionnaire and video data, we were able to confirm our hypothesis and suggested that children in different learning stages are more motivated with robots with different roles during practicing. This finding is in line with self-concept development in instrument learning (Greenberg, 1970) and social facilitation theory (Zajonc, 1965). That is, beginners have not developed clear acknowledgment about their abilities, which may have a negative impact on their motivation; a positive audience might encourage them better than an evaluative judge. Additionally, beginners do not yet have the (evaluation) skills to estimate which evaluation information from others can be used. As for the advanced players, their music self-concepts have been greatly reinforced by earlier music experiences, which makes them conscious of their own ability (Ireson and Hallam, 2009). In other words, advanced players usually have a higher self-concept. Without companionship, it is easy for them to lose patience in instrument practice. The encouragement and feedback provided by the robot may reinforce the self-concept of the advanced players (Greenberg, 1970; Bong and Clark, 1999; Lamont, 2011), which further improved their motivation in practicing. Combined with our empirical results, we believe that applying different roles of the robot in different learning stages would help children keep motivated in music instrument learning. More specifically, we suggest employing social robots in children’s instrument learning, especially for the advanced players. Also, it seems better to use less evaluative robot for the beginners who just started learning a musical instrument. At the same time, we suggest that individual characteristics of learners during piano practicing might require more complex pedagogical approaches.

In addition, results from the observations further strengthened our confidence to confirm the conclusion. Within all the indicators that we coded for motivation, the most important result is that robot roles have an impact on children’s persistence in different learning stages. According to the definition of motivation as a “process whereby goal-directed activity is energized and sustained” (Pintrich and Schunk, 2002), persistence is a key indicator of sustainment. This result aligns with the questionnaire result, which showed advanced players had better persistence with a robot than alone and beginners tend to have better persistence with the nonevaluative robot.

While the main results were in accordance with our expectations, the exploratory results also suggested that gender might be a determinant of a user’s evaluation of the robot, in interaction effect with the robot’s role. A potential explanation might be that the interaction between gender and robot role influenced children’s motivation through persistence in music practice. However, in the review about gender differences in self-concept with children and adolescents done by Wilgenbusch and Merrell (1999), female participants reported significantly higher levels of self-concept in the musical domain among elementary grade participants (grade 1–6). Applying this finding to our case, girls should persist longer with the evaluative robot, which contrasts with our result. Still, Wilgenbusch and Merrell (1999) also indicated that their results were based on quite small effect sizes. And they also found that males reported significantly stronger self-concepts in the musical domain among secondary grade participants (grade 7–12). Our participants consist of children in both of their grade groups, and therefore, further work needs to be carried out to establish whether gender is a crucial factor that can affect children’s motivation in the child–robot interaction.

Although our findings indicated the idea that different roles of the robot should be employed in different stages of children’s instrument learning, we are aware that our research may have two limitations and which future studies can extend. The first is about the appearance and function of the robot. We used the SocibotMini, which does not have arms and cannot move. These features limited the possibility of interactions and may cause distrust from children on robot’s music-related abilities. Furthermore, this robot requires real-time operation from the controlling system to be able to react. However, it was impossible for the researcher to eliminate the delay in the interaction between children and the robot, even with a real-time surveillance camera. Therefore, there should be an inevitable influence on the interaction. This can be improved by using a full-body robot with an intelligent system. Secondly, even though we investigated child–robot interaction design for long-term companions with different learning stage groups, another perspective is studying changes in learning styles within one individual. For example, with the children’s grows, their perception and preference would change. It is important to investigate the link between children’s age and robot role preference in a long-term interaction design. Furthermore, instead of using children in different learning stage groups, future experimental investigations are needed to find out children’s perception and preference of the robot roles within the development of the same individual. By then, we may focus on discovering the long-term effect of social robots in music instrument learning. In future research, it is also crucial to investigate more detailed factors that can affect children’s motivation and performance in instrument learning, for instance, find out the impact of different kinds of evaluation in the instrument learning context (Sasfy and Okun, 1974). Another interesting research direction to investigate would be music-induced emotions. Emotion experiences are valid indicators for motivation (Csikszentmihalyi, 2000; Pekrun, 2006) and emotion expressions (e.g., facial expressions) are measurable elements of people’s emotion experience. In the context of instrument learning, some of the basic indicators (e.g., smile) are rather rare in instrument learning and practice. However, not showing a smile is not equal to not being happy, as the expressions of positive emotions may be hidden in the instrument learning context. In this case, it is valuable to investigate whether it is correct to use common indicators to measure motivation in instrument learning.

In conclusion, our study confirmed the impact of different robot roles in children’s instrument learning process, which offers more insight into child–robot interaction and robot role design in musical instrument learning. Specifically, our findings shed light on personalization in HRI, that is, adapting the role of the robot to the characteristics and the development level of the user.

## Data Availability

The raw data supporting the conclusion of this article will be made available by the authors, without undue reservation.
